# Refractive astigmatism in phaco-canaloplasty vs phaco-non-penetrating deep sclerectomy

**DOI:** 10.1038/s41598-022-12451-7

**Published:** 2022-05-21

**Authors:** Anna Byszewska, Jacek Rudowicz, Katarzyna Lewczuk, Joanna Jabłońska, Marek Rękas

**Affiliations:** Ophthalmology Department, Military Institute of Medicine, Warsaw, Poland

**Keywords:** Diseases, Health care, Medical research, Optics and photonics

## Abstract

This study aimed to assess refractive astigmatism, in phaco-canaloplasty (PC) vs phaco-non-penetrating deep sclerectomy (PDS) in a randomized, prospective study within 24 months. Patients were randomized pre-operatively, 37 underwent PC and 38 PDS. The following data was collected: BCVA, IOP, number of antiglaucoma medications, refraction with autokeratorefractometry. The assessment of astigmatism was simple arithmetic and vector analysis (calculations included cylinder with axis in form of centroids) and included double angle plots and cumulative refractive astigmatism graphs. Pre-operative mean BCVA in PC was 0.40 ± 0.43 and was comparable to BCVA in PDS 0.30 ± 0.32logMAR (P = 0.314). In the sixth month follow-up, mean BCVA showed no difference (P = 0.708) and was 0.07 ± 0.13 and 0.05 ± 0.11, respectively. However, 2 years after the intervention mean BCVA was better in PC 0.05 ± 0.12 than in PDS 0.12 ± 0.23 and it was statistically significant (P = 0.039). Mean astigmatism in PC at baseline was 1.13 ± 0.73Dcyl, at 6 months it was 1.09 ± 0.61 and at 2 years 1.17 ± 0.51. In PDS at baseline 1.35 ± 0.91 at 6 months 1.24 ± 0.86 and at 2 years 1.24 ± 0.82. There were no differences between the groups in mean astigmatism throughout the study. Centroids (mean of a cylinder with axis) in PC were pre-operatively 0.79D@172˚ ± 1.10Dcyl, at 6 months 0.75D@166˚ ± 1.01 and at 24-months 0.64D@164˚ ± 1.11 and in PDS pre-operatively 0.28D@10˚ ± 1.63D at 6 months 0.26D@11˚ ± 1.5 and at 24-months 0.47D@20˚ ± 1.43. The direction of mean astigmatism was against the rule in all analyzed time points. The mean baseline IOP in PC was 19.4 ± 5.8 mmHg and 19.7 ± 5.4 mmHg in PDS(P = 0.639). From the 6-month IOP was lower in PC, at 24-months it was 13.8 ± 3.3 mmHg in PC and 15.1 ± 2.9 mmHg in PDS(P = 0.048). In both groups preoperatively patients used median(Me) of three antiglaucoma medications(P = 0.197), at 24-months in PC mean 0.5 ± 0.9 Me = 0.0 and 1.1 ± 1.2 Me = 1.0 in PDS(P = 0.058). Both surgeries in mid-term observation are safe and effective. They do not generate vision-threatening astigmatism and do not even change the preoperative direction of mean astigmatism. Refractive astigmatism is stable throughout the observation.

## Introduction

Nowadays glaucoma surgery is under constant innovation. Gold standard—trabeculectomy is the most often performed glaucoma procedure worldwide^1^, however, is associated with certain risks, due to its’ perforating character. Well-known complications such as postoperative hypotony, macular edema, hyphema, shallow anterior chamber, or filtering bleb associated problems such as avascularity, late bleb leakage leading to infections or bleb insufficiency, cause qualifications for surgeries in more advanced stages of glaucoma. The ratio risk of the surgery to benefit is relatively high to perform procedures in early and moderate glaucoma patients.

Then the non-penetrating procedures were developed. Non-penetrating deep sclerectomy although not such IOP lowering as compared to trabeculectomy, enables a much better safety profile with avoidance of most of the serious, sight-threatening complications. Its safety and efficacy are thoroughly described in the literature^2,3^.

Canaloplasty is a relatively new procedure in glaucoma surgery, introduced in scientific publications for the first time in 2007 by Lewis^4^. It represents one step further in safety and quality of life for a glaucoma patient. It is also a non-penetrating procedure and the surgical approach compromises of similar steps as DPS, up to the dissection of the trabeculo-Descemet’s membrane^5^. Both procedures, although technically challenging for surgeons, enabled a change of attitude in referring patients for glaucoma surgeries in early and moderate stages.

We conducted a prospective randomized study of PC and PDS with a 24-months postoperative follow-up.

Although both surgeries are non-penetrating, there are many differentiating factors intra and postoperative which may influence the postoperative refraction. During PC cautery is used as little as possible, to spare the outflow vessels, while it is not that important in PDS- this may vastly change the architecture of the wound. In PDS the outflow depends on well-functioning filtering bleb, to achieve that only two loose fixating sutures are used on the superficial scleral flap, whereas in PC there are five tight sutures and the bleb is aimed to be watertight and flat. Additionally, in PDS to maintain outflow though the bleb , we used 5-fluouracil (5-FU) in subconjunctival injections. This substance may cause transient irregular astigmatism, due to toxicity to the corneal epithelium. And last but not least in PC a prolene suture is inserted and tightened inward in Schlemm’s canal. Such tension changes the anatomical relations, which was already shown with ultrabiomicroscopy (UBM) or anterior segment OCT.

In this paper, we want to concentrate on the refractive aspects of both non-penetrating surgeries. Refractive astigmatism may be considered as one of the safety indicators, as it directly influences visual acuity, and quite frequently a correction with glasses is poorly tolerated.

This analysis aimed to describe the 24-month postoperative course of astigmatism for both procedures. The period in which postoperative astigmatism stabilizes is still under research. Cunliffe et al.^6^ showed data 2 months after penetrating procedures, Dietze et al.^7^ after 3 months, Willekens et al.^8^ observed stabilization after 3 months, Claridge et al.^9^ and Hong et al.^10^ reported changes up to 12 months. Many authors suggested the need for long-term observation. In this paper we analyze preoperative data, then after 6 and 24 months.

To our knowledge, there is little literature describing astigmatic changes after glaucoma procedures, and none in 24 months period concerning canaloplasty and PDS, compared to other aspects of glaucoma surgery.

## Subjects and methods

### Subjects

This paper represents further aspects of a previously published study, where patients and methods were described in detail^5,11^. The methods and results are presented in line with Guidelines on Design & Reporting Glaucoma Trials^12^. This study is in line with European Union entitled Good Clinical Practice for Trials on Medical Products in the European Community and the tenets of the World Medical Association Declaration of Helsinki. The project received approval from the Institutional Review Board of the Military Institute of Medicine in Warsaw (9/WIM/2011) and was registered at https://www.clinicaltrials.gov NCT01726543 on 15/11/2012.

Patients with coexisting glaucoma and cataract (NC1 and NC2) classified according to the Lens Opacificities Classification System LOCS III were qualified for the study. In all cases glaucoma procedure was performed with cataract extraction and monofocal IOL implantation.

### Glaucoma types

Types of glaucoma eligible for the study were primary open-angle glaucoma (POAG), pseudoexfoliation glaucoma (PEX), and pigmentary glaucoma. Apart from the type of glaucoma, patients must have had at least one of the following features: well-documented progression of the visual field; non-compliance in anti-glaucoma therapy or allergy to topical medications, daily fluctuations in pressure. Both PC and PDS as well as surgical alternatives were explained in detail to candidates. After declaring willingness to participate in the study, each patient signed an informed consent.

### Design

The design of the study was randomized and prospective. Randomization into groups was carried out by a random sorting algorithm with an allocation ratio set to 1.0 on the day of surgery.

A single physician (AB) was responsible for the preoperative examination, randomization, and postoperative care. Also, a single surgeon (MR) performed all the surgical procedures and then was excluded from any further medical involvement in this study to avoid bias.

In the course of the study the intraocular pressure (IOP), the number of antiglaucoma medications, Best Corrected Visual Acuity (BCVA), autokeratorefractometry (AKR), Humphrey 24-2 visual fields (VF), Optical Coherent Tomography (OCT) morphology of the surgical site, Quality of life (QoL) assessment- were collected prospectively^5,11^.

### Preoperative examination

At the baseline visit, ophthalmic and general medical history was taken. During the visit the AKR measurement was carried out as the first examination, to avoid any influence from other, especially contact examinations. Then uncorrected distance visual acuity and BCVA were checked, followed by parameters required for IOL calculation such as axial length and keratometry. Central corneal thickness was also evaluated. We collected data of IOP with the number of antiglaucoma medications taken. Routinely a gonioscopy was performed. All subjects had a dilated slit-lamp examination.

### IOP measurements

The IOP was measured with a Goldmann applanation tonometer and all measurements included in the analysis were taken between 8 and 10 am. During the qualification visit, a diurnal curve of IOP was assessed and a single measurement on the day of surgery was taken. Routinely, two measurements were taken, if they varied more than 1 mmHg, the third one was taken, and the outcome was the average of the three measurements. Based on IOP values, the course of mean IOP was defined.

### BCVA

A standard ETDRS chart was used to measure the BCVA. The calculations were performed using logMAR -a logarithm of the minimum angle of resolution. SRK T formula was used to calculate the IOL.

### Surgical procedure

Surgical procedures were previously described in detail^5,11^^.^ For a better understanding of the outcomes, the description is cited below. All surgical procedures were performed under retrobulbar anesthesia (2% xylocaine and 0.5% bupivacaine) by one experienced surgeon (MR). Classic canaloplasty was carried out with a standard canaloplasty set (iTrack from Ellex Medical Lasers Pty Ltd., Adelaide, Australia). Nonpenetrating deep sclerectomy was carried out with a Healaflow implant, a slowly resorbable crosslinked viscoelastic gel (Anteis Ophthalmology, Geneva, Switzerland). In both procedures, a fornix-based superficial scleral flap was dissected, followed by a deep scleral flap and a TDM dissection. During the next step, a 2.2 mm clear corneal temporal incision was made, the cataract was phacoemulsified (Infiniti Vision System, Alcon Surgical, Fort Worth, TX), and a monofocal IOL was implanted. The deep scleral flap was excised. In PC, 360° of the Schlemm’s canal circumference was catheterized and viscodilated with 10.0 Prolene suture left under tension to distend the trabecular meshwork inward. In PDS, after dissection of TDM, the roof of Schlemm’s canal was removed. The superficial scleral flap was then loosely sutured to the sclera, and HealaFlow was injected under the flap to create a filtering bleb. In PC, the superficial flap was sutured tightly to prevent leakage and subsequent bleb formation with interrupted 10-0 monofilament nylon suture. The conjunctiva was sutured down over the limbus with one interrupted 8.0 Vicryl suture.

### Postoperative protocol

The postoperative visits were scheduled for days 1 and 7 and 1, 3, 6, 12, 18, and 24 months after surgery. However, patients were informed that they can show up any time when needed. A topical steroid and antibiotic combination was prescribed for 4 weeks after surgery. During postoperative examinations, AKR data was collected as the first one, followed by BCVA assessment and then IOP was determined and the number of hypotensive medications was noted. Afterwards the anterior segment examination including gonioscopy (in the case of PC, to assess any possible complications) and at the end of visit a dilated pupil fundoscopy was performed. Glaucoma drugs were discontinued on the day of surgery. In the course of the study, medications were administered again, when required, under the guidelines of the European Glaucoma Society. Complications that occurred within 30 days were analyzed as early, whereas after 30 days were considered as late. To keep IOP at a sufficiently low level, additional procedures were carried out. In the case of PDS, they were associated with filtering bleb maintenance such as 5-FU subconjunctival injections (when signs of bleb failure were noticed—new, tortuous vessels, hyperemia, or encapsulation), suture lysis, and needling. 5-FluoroUracil was injected in a dose of 0.2 ml (5 mg) in the lower fornix of the operated eye. If needling of filtering bleb was required (encapsulated and flat blebs, which caused elevated IOP), the patient was anesthetized with proxymetacaine eye drop, and needling was performed at the slit lamp, followed by 5-FU injection. Goniopuncture, which is laser puncture of TDM, was performed in both PC and PDS, when filtration through TDM was suspected to be insufficient (with an Nd: YAG laser, about 3–20 shots were applied using energy ranging from 2 to 4 mJ).

### Refractive parameters

The refraction was recorded at the central 3-mm diameter by Auto-kerato-refractometry (Topcon TRK 2P), which is serviced according to manufacturer recommendations. The AKR data was analysed at the baseline, then 6 months and 24 months post-surgery. All calculations were performed after the transposition of the cylinder values to the plus form.

Two types of analysis were performed- refractive and vector.

### Arithmetic

The first one is the simple arithmetic calculation of the mean of the cylinder, without considering its axis. This analysis was performed to discuss the numerical results available in the literature. Such a difference in the mean value of the cylinder allows reporting the mean change in the magnitude of astigmatism. Aggregate data of astigmatism values are depicted in the cumulative data plots.

### Vector

The vector analysis—the second method presented is a calculation of the cylinder change with consideration of its axis, which results in a calculation of centroid. The preoperative and postoperative refractive measurements (cylinder with its axis) were evaluated by vector analysis, according to the method proposed by Holladay et al^13^ Centroid is a form of astigmatism, which is calculated in a certain way (see below), so that it can be presented as a set of x and y values on a cartesian graph (standard polar data are converted to cartesian values suitable for calculations, but still including the axis). What is most important centroid incorporates the magnitude and axis of astigmatism. It can be then included in the standard descriptive statistics (means, standard deviations). When a larger set of data is presented, centroid means the mean of all values, and a trend for astigmatism can be described (with the rule, against the rule, or oblique).

The data were converted from standard polar values (cylinder and axis) to Cartesian values (point with x,y coordinates) to evaluate trends in astigmatism (against the rule, with the rule or oblique) and to define mean astigmatism in centroid form.

For conversion from polar to cartesian values the following mathematical formula was applied:

$$\begin{gathered} {\text{x}} = {\text{cyl}}*{\text{cos}}\left( {{2}*{\text{axis}}} \right) \hfill \\ {\text{y}} = {\text{cyl}}*{\text{sin}}\left( {{\text{2axis}}} \right) \hfill \\ \end{gathered}$$

Then the Cartesian coordinates were converted to standard polar values, with formulas:

$$\begin{gathered} {\text{cyl}} = \surd \left( {{\text{x2}} + {\text{y2}}} \right){\text{Angle}} = {1}/{2 }*({\text{ tan}} - {1 }\left( {{\text{ y }}/{\text{ x }}} \right) \hfill \\ {\text{if x}} > 0{\text{ and x}} > 0{\text{ angle }} = {\text{ axis}} \hfill \\ {\text{if x}} < 0 {\text{ axis}} = {\text{angle }} + {9}0^\circ \hfill \\ {\text{if x}} > 0{\text{ and y}} < 0{\text{ axis}} = {\text{angle }} + { 18}0^\circ \hfill \\ {\text{if x}} = 0 {\text{ a y}} < 0{\text{ axis}} = {135}^\circ \hfill \\ {\text{if x}} = 0 {\text{ a y}} > 0{\text{ axis}} = {45}^\circ \hfill \\ {\text{if x}} = 0{\text{ i y}} = 0{\text{ axis}} = 0^\circ \hfill \\ {\text{if y}} = 0{\text{ a x}} < 0{\text{ axis}} = {9}0^\circ \hfill \\ {\text{if y}} = 0{\text{ a x}} > 0{\text{ axis}} = 0^\circ \hfill \\ \end{gathered}$$

The calculation was performed for each individual, to compute individual surgically induced refractive change. The mean of all x and y allowed to calculate aggregate refractive change for the analyzed groups.

The data was displayed in the double angle plots (the angles had to be doubled as the astigmatism vector returns to the same value when it traverses an angle of 180 degrees).

The major and minor axes of the ellipse surrounding the centroid were determined by standard deviations of x and y coordinates. The trend of astigmatism was evaluated depending on centroid values and axis.

The double-angle plots were depicted with the help of a double-angle plot tool for astigmatism available on the ASCRS website^14^.

### Statistical analyses

The Shapiro–Wilk was used for the assessment of the normality of the data. Non-parametric data were calculated with a χ^2^ test with corrections. Comparisons between the groups (IOP, BCVA, refractive astigmatism) were performed with U Mann–Whitney test, and the Student's T-test. Friedman's analysis of variance (ANOVA) for matched groups, mean ranks, and rank sums were also used for posthoc comparisons. A *P*-value of 0.05 or less was considered significant. Calculations were performed using the Statistica 10.0 PL software.

## Results

### Subjects

Throughout the study, 37 patients were randomized for PC and 38 for PDS. All patients were Caucasian. Mean age was comparable for both groups, in PC was 75.1 ± 8 years and 73.6 ± 6.2 years (P = 0.079). The sex structure and side of the surgery did not differ between both arms of the study. The glaucoma types were primary open-angle and pseudoexfoliation glaucoma (27/10 in PC and 34/4 in PDS), P = 0.124 (Table 1).

**Table 1:** Patients’ demographic data.

	Phacocanaloplasty	Phaco-deep sclerectomy	P-value
Data	Mean ± SD ratio		
N	37	38	0.908 ∗
Age (years)	75.1 ± 8.1	73.6 ± 6.2	0.079^#^
Sex (female/male)	15/22	21/17	0.202 ∗
Eye (right/left)	15/22	17/21	0.713 ∗
Glaucoma type: POAG/PEX	27/10	34/4	0.124 ∗

^∗^Chi^2^.

^#^Mann–Whitney U.

### BCVA

The BCVA data is presented in the logMAR scale. Baseline BCVA in the PC and PDS group was comparable and was 0.40 ± 0.43 and 0.30 ± 0.32 , respectively (P = 0.314). After 6 months visual acuity improved significantly to 0.07 ± 0.13 and 0.05 ± 0.11 (P = 0.708). A 24-month follow-up revealed a better mean BVCA in PC, which was 0.05 ± 0.12 than in PDS 0.12 ± 0.23 (P = 0.039). For both groups improvement was significant in all-time points during the study (P < 0 001). Two years after surgery, stable vision or improvement of one or more Snellen lines was present in all patients after PC and in the majority of PDS (85.3%). A decline of one line was noted in three PDS (8.8%) patients. The decline of two lines was observed in two subjects (5.9%). One developed diabetic macular edema and the second had transient visual acuity instability, as the good vision was regained at the following examination (0.0 logMAR). No changes in BCVA were observed in 8.8% PDS and 13.3% PC eyes.

### Astigmatism

Refractive data was available from 35 PC and 37 PDS patients. Mean astigmatism at the baseline in PC was 1.13 ± 0.73 Dcyl, whereas in PDS 1.35 ± 0.91 Dcyl, at 6 months post-surgery it was stable 1.09 ± 0.61 Dcyl in PC and respectively 1.24 ± 0.86 for PDS. At the end of the 24-month observation, it was still around one Diopter and precisely for PC 1.17 ± 0.49 Dcyl and 1.24 ± 0.82 Dcyl for PDS. Throughout the whole observation, there were no differences found between the studied groups in mean astigmatism. Also, Friedman ANOVA analyses showed no statistical differences within the groups during this 24 months timespan (Table 2).

**Table 2:** Arithmetic mean of astigmatism.

Dcylinder_month	N	Mean [Dcyl][minimum; maximum]	SD	Median	1st, 3rd quartile	N	Mean [Dcyl][minimum; maximum]	SD	Median	1st, 3rd quartile	U-Mann–Whitney test p-value
	PC					PDS					
Dcyl_0	35	1.13 [0; 3]	0.73	1.0	[0.5; 1.5]	37	1.35 [0.25; 4]	0.91	1.25	[0.75; 1.5]	P = 0.544
Dcyl_6	34	1.09 [0; 3.5]	0.61	1.0	[0.5; 1.25]	37	1.24 [0; 3.95]	0.86	1.0	[0.75;1.5]	P = 0.595
Dcyl_24	31	1.17[0;2.75]	0.49	1.0	[0.75;1.5]	37	1.24 [0; 3.5]	0.82	1.25	[0.75;1.75]	P = 0.917
Friedman_ANOVA p-value		Chi^2^ ANOVA (N = 32, df = 2) = 1.407407 P = 0.49475					Chi^2^ ANOVA (N = 36, df = 2) = 0.5909091 P = 0.74419				

Results of statistical analysis between the groups (U-Mann Whitney Test) as well as for separate groups in analyzed time points (Friedman ANOVA). Statistical significance for P < 0.05.

**Figure 1:**
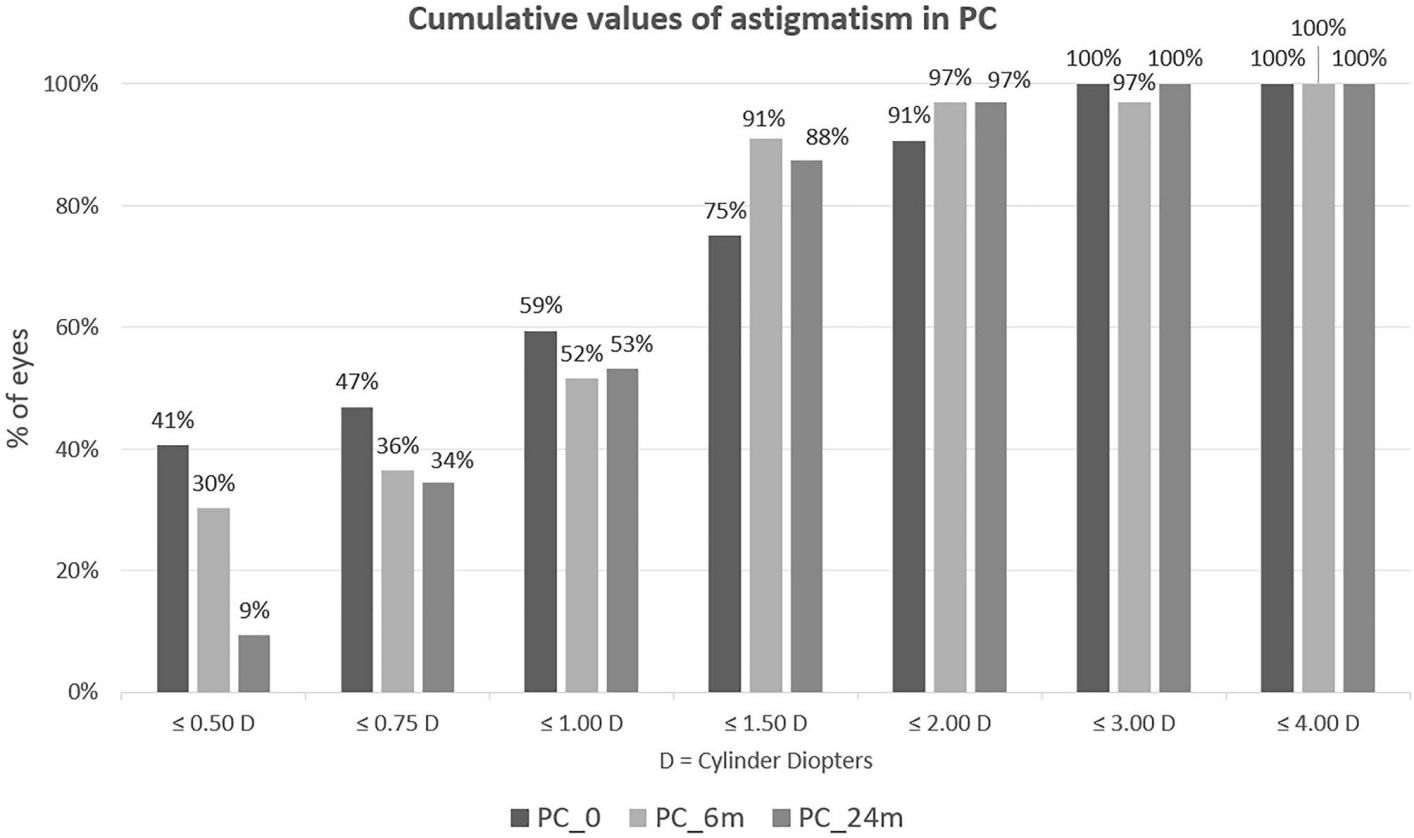
Cumulative astigmatism in PC.

**Figure 2:**
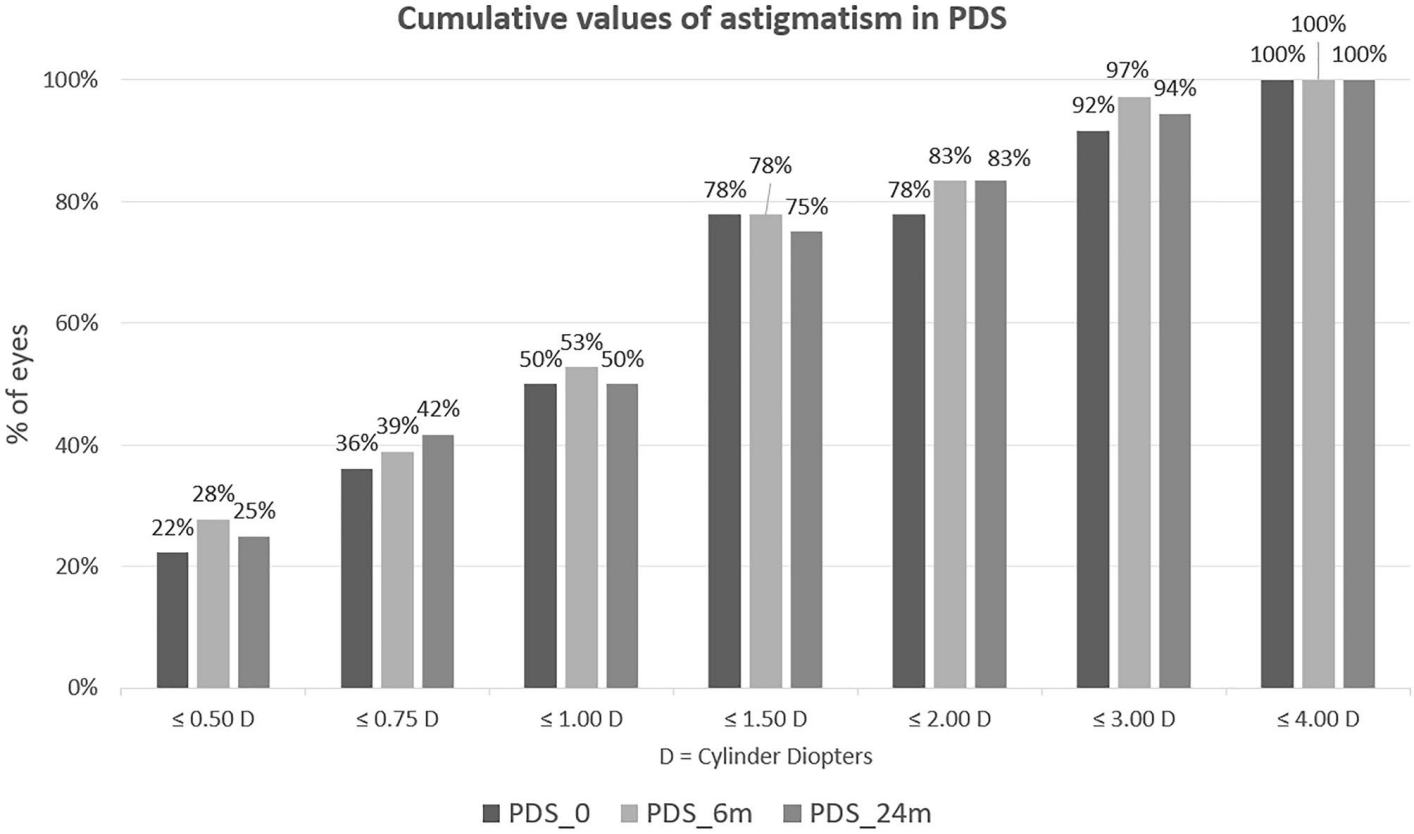
Cumulative astigmatism in PDS.

Cumulative astigmatism data is depicted in the diagrams (Figs. 1, 2). At baseline, 47% of the PC patients and 36% of PDS had astigmatism ≤ 0,75D. Six months postoperatively in both groups about half of the patients had astigmatism ≤ 1D, whereas ≤ 1,5 D, 91% of PC, and 78% of DS patients. At the end of observation, almost all of the subjects had astigmatism lower than 2D, whereas in DS there was 6% of patients who had astigmatism higher than 3D.

### Vector analysis results

**Figure 3:**
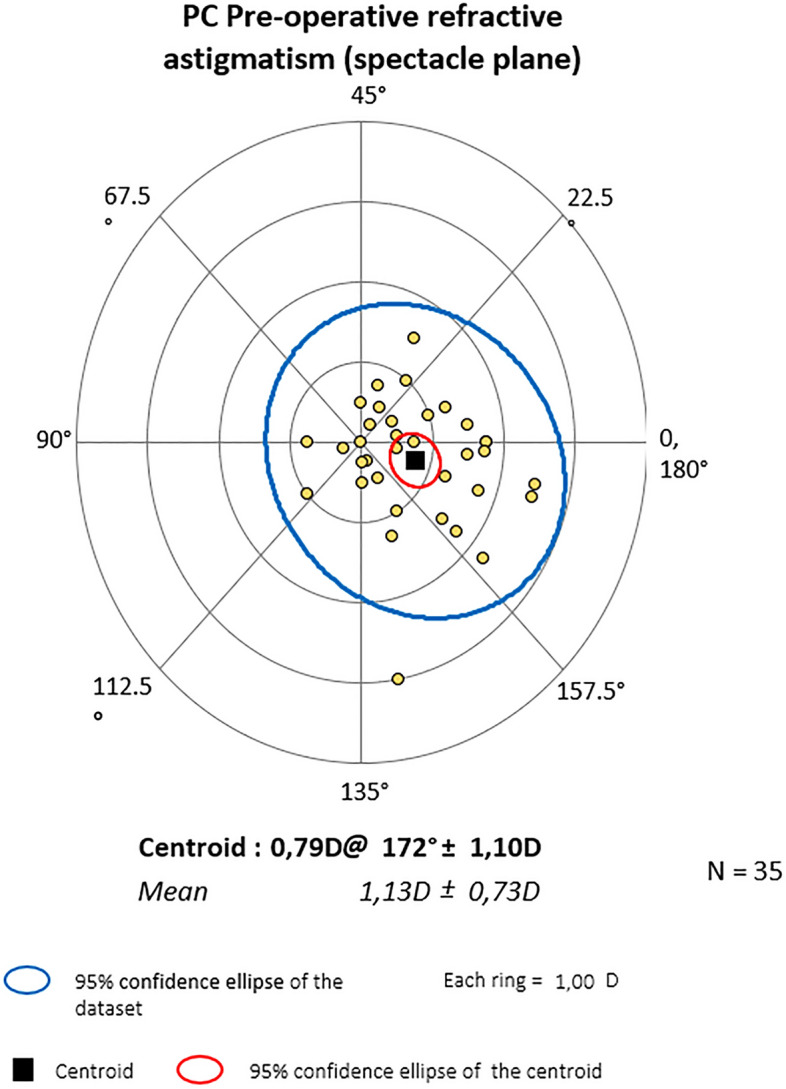
Preoperative astigmatism on the double angle plot in PC. The trend is ATR. The yellow dots represent a single subject’s data.

**Figure 4:**
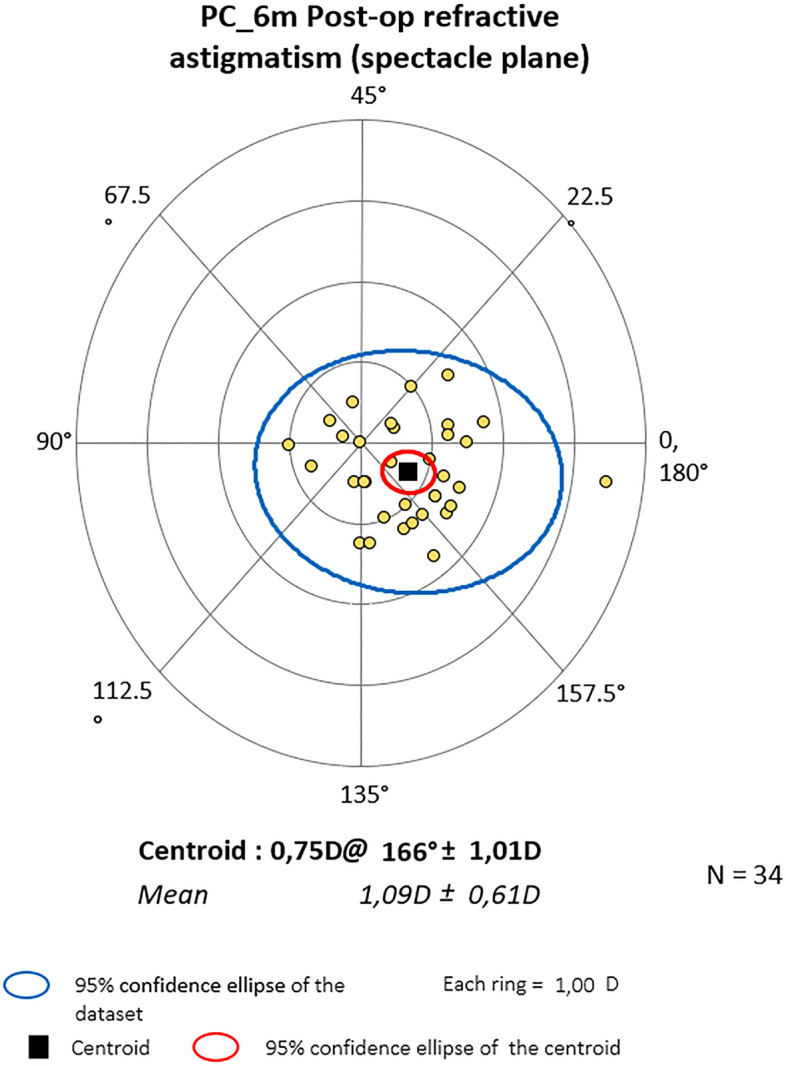
Astigmatism on the double angle plot 6 months post-op in PC. The trend is ATR. The yellow dots represent a single subject’s data.

**Figure 5:**
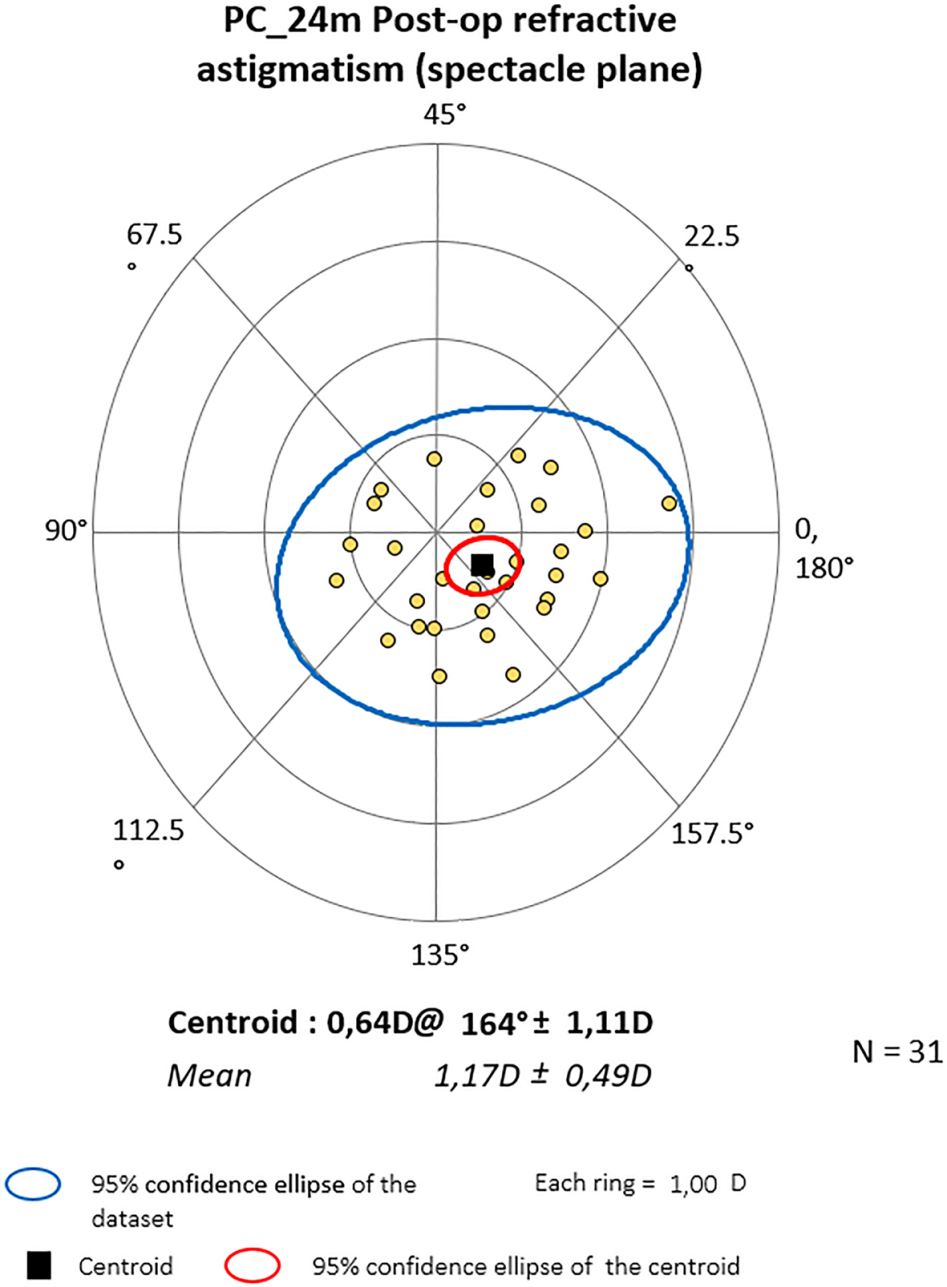
Astigmatism on the double angle plot 24 months post-op in PC. The trend is ATR. The yellow dots represent a single subject’s data.

**Figure 6:**
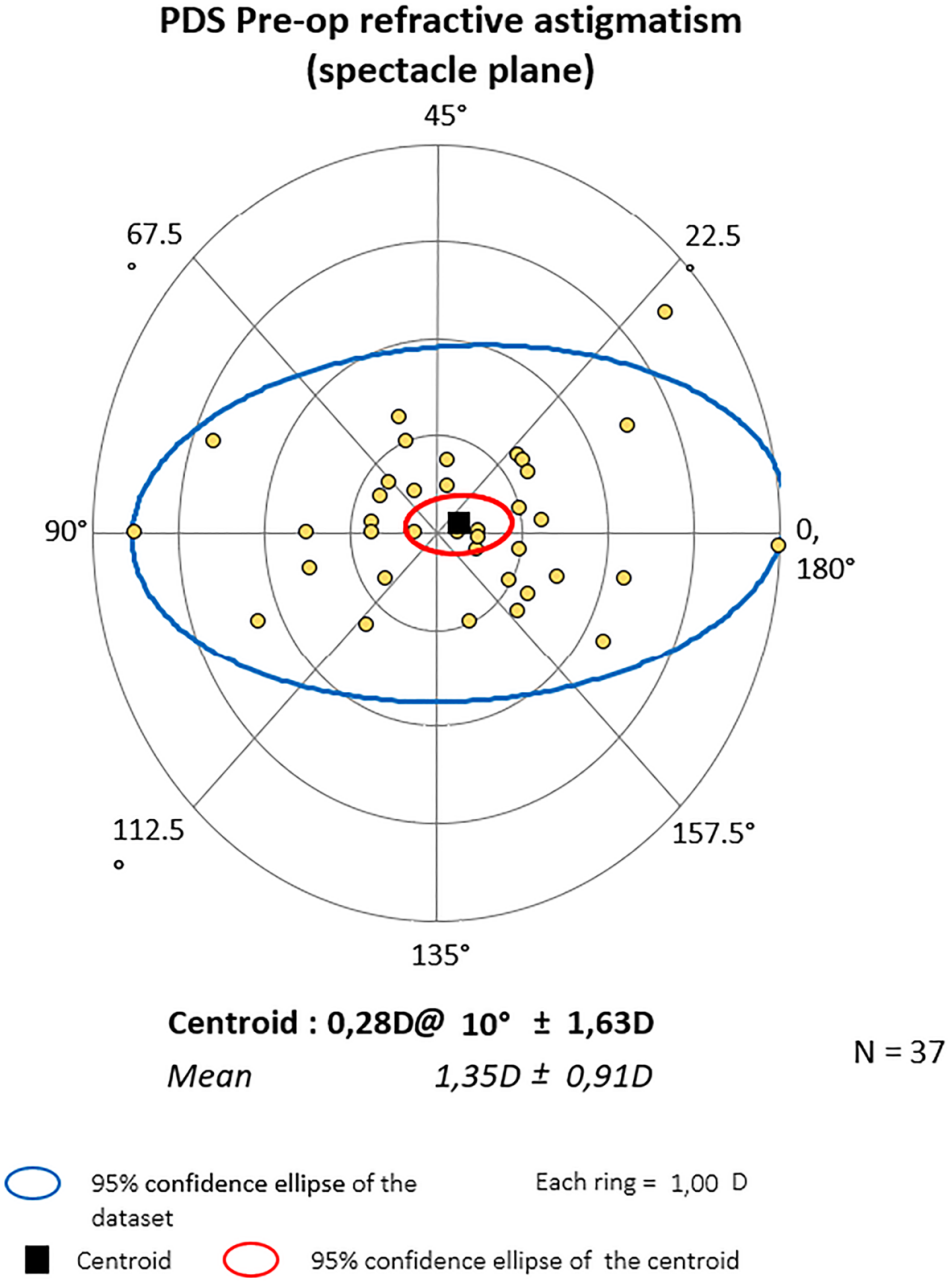
Preop astigmatism on the double angle plot PDS. The trend is ATR. The yellow dots represent a single subject’s data.

**Figure 7:**
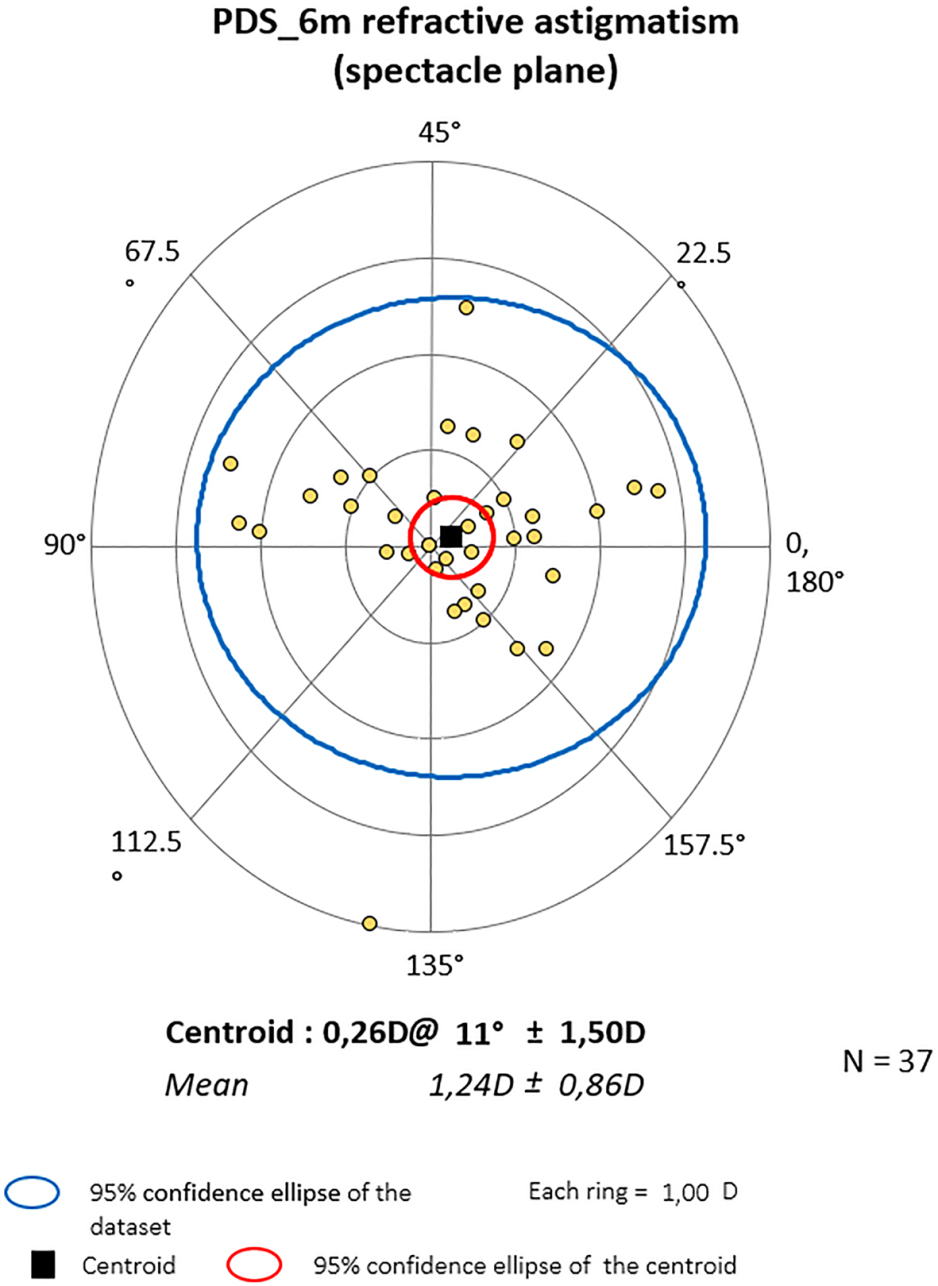
Astigmatism on the double angle plot 6 months post-op in PDS. The trend is ATR. The yellow dots represent a single subject’s data.

**Figure 8:**
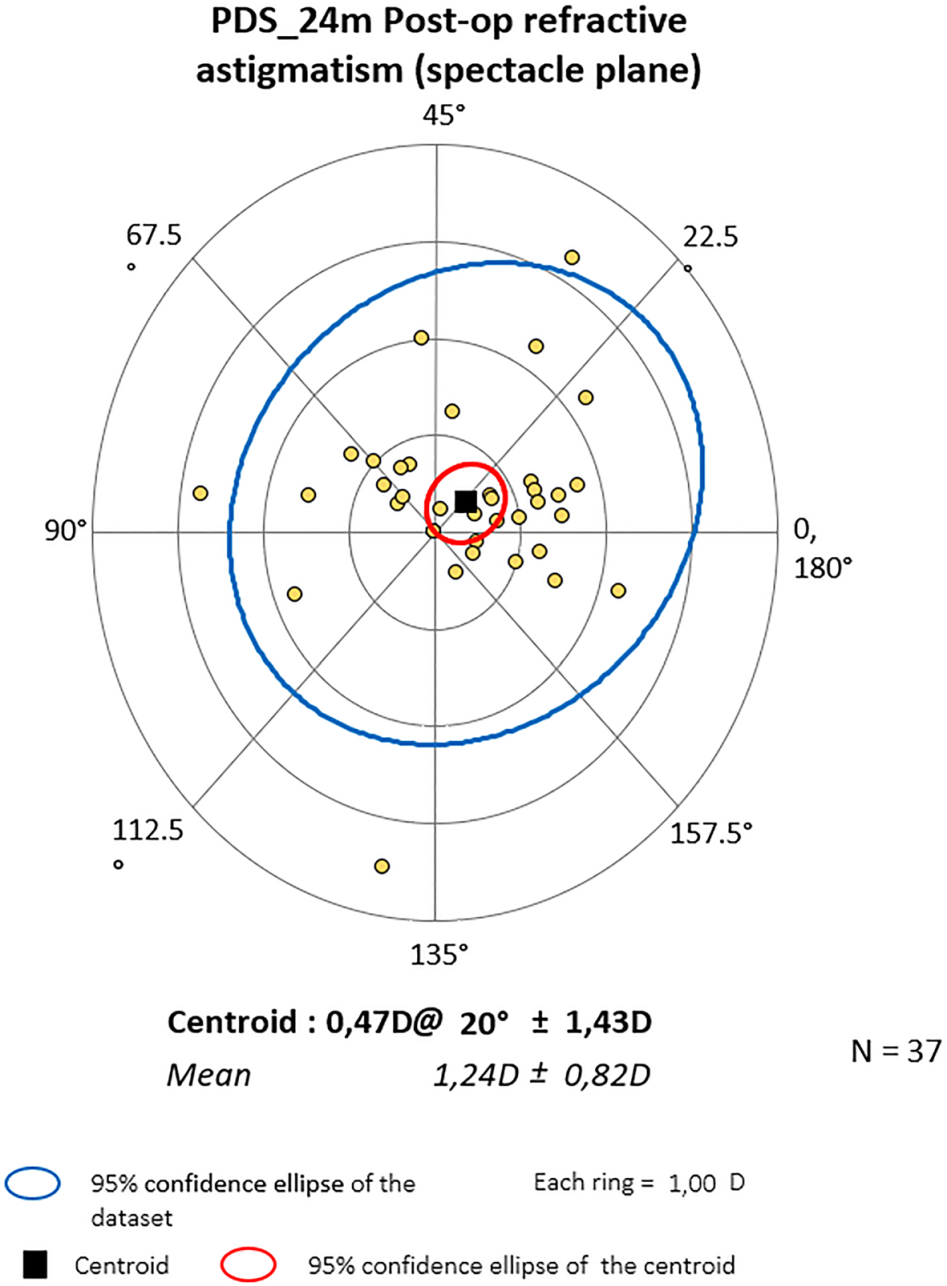
Astigmatism on the double angle plot 24 months post-op in PDS. The trend is ATR. The yellow dots represent a single subject’s data.

Mean centroid before the surgery was 0.79D @172˚ ± 1.10 for PC and 0.28D @ 10˚ ± 1.63 for PDS (P = 0.364). Six months later it was 0.75D@166˚ ± 1.01 and 0.26D@11˚ ± 1.50 respectively (P = 0.828). At the end of observation mean astigmatism in PC was 0.64D@164˚ ± 1.11 and 0.47D@20˚ ± 1.43D in PDS (P = 0.874). Also, Friedman_ANOVA analyses for separate groups showed no statistical differences within study time. The centroids are depicted graphically in double-angle plots below. In both groups, preoperative astigmatism was against the rule (ATR) and the trend was stable throughout the study. (Figs. 3, 4, 5, 6, 7 and 8).

### IOP

The mean baseline IOP in PC was 19.4 ± 5.8mmHgand 19.7 ± 5.4 mmHg in PDS and did not differ between the groups (P = 0 639). Within the first 3 post-op months, no difference in mean IOP was found. Starting from the 6th month, the mean IOP was lower in PC, and the difference lasted until 24 months (P = 0.048). After 6 months mean IOP for PC was 13.02 ± 3.24 and in PDS 14.2 ± 2.9. At the end of observation, mean IOP significantly decreased to 13.8 ± 3.3 mmHg (P = 0.001) and 15.1 ± 2.9 mmHg (P = 0.001), respectively. Mean IOP was reduced by 25.7% in PC and 18.9% in PDS.

### Medications

Fewer medications were used after surgery than before in both groups (P < 0 05). Baseline mean number of meds in PC was 2.6 ± 0.9, Me = 3.0, and in PDS, 2.9 ± 0.9, Me = 3.0 (P = 0.197). After 6 months in PC it was 0.2 ± 0.5 ME = 0 and for PDS 0.2 ± 0.6 ME = 0 (P = 0.639). At the 24 months follow up it was still without statistically significant difference in PC 0.5 ± 0.9 Me = 0.0 and 1.1 ± 1.2 Me = 1.0 (P = 0.058).

## Conclusion/discussion

We found only one paper describing astigmatic changes in canaloplasty. Moelle et al.^15^ observed 26 canaloplasty patients and the mean preoperative astigmatism was 0.77 ± 0.5 D, then it was higher in the initial healing phase to reach stabilization at 6 months (0.86 ± 0.52D). There was no significant difference in preoperative astigmatism and astigmatism at 6 months (P > 0.05). Findings are similar to those presented in this paper.

Egrilmez^16^ assessed astigmatism in 10 patients following deep sclerectomy and 12 after viscocanalostomy and confronted this data with trabeculectomy results. The lowest arithmetic averages of induced vectors were in viscocanalostomy, then deep sclerectomy, being both statistically lower at 3 and 6 months compared to trabeculectomy. In our study arithmetic averages are stable within the groups throughout the study. Preoperatively the mean astigmatism value was 1.13 ± 0.73 in PC and 1.35 ± 0.91 in PDS and it stayed on a similar level throughout the study, which is confirmed in Friedman ANOVA analysis.

On the contrary, El-Saied^17^ compared a relatively large group of trabeculectomy and deep sclerectomy subjects and concluded that both after 6 months induce significant postoperative astigmatism, due to flattening along the vertical meridian, more with trabeculectomy than deep sclerectomy.

Vector analysis allowed us to access the trend of astigmatism. In both groups throughout the study, it was against the rule. As it was against the rule also preoperatively we are not trying to look for the causes of the postoperative trend, as the surgery did not significantly influence the trend. And such a trend describes the studied population.

In our paper, both analyzed procedures, although non-penetrating, characterize certain differences. The principle of canaloplasty is enhanced outflow through distal outflow pathways-dilated Schlemm’s canal, collector channels^18,19^, while dissection of the scleral flap is performed to get access to Schlemm’s canal. On the other hand, NPDS functioning is based to a vast degree on the subconjunctival outflow, apart from transscleral and suprachoroidal pathways^20^.

Nonetheless, the presence of the scleral flap itself and the quantity and strength of suturing may affect the direction of postoperative astigmatism. In the analyzed PDS group there are only two scleral sutures on the superficial scleral flap, one on each side, which allows a controlled flow of aqueous humor to subconjunctival space. The flap is sutured loosely. In PC, on the contrary, five tight sutures (one to the apex and two to each side) were put to secure the watertight closure of the flap.

In neither of the procedure, a full-thickness scleral gap was dissected, the partial thickness of scleral flaps was comparable in both groups. The layer of the trabeculoDescemet’s window enabled the uninterrupted continuity of the tissues, which may also contribute to lesser change in refraction^16^.

The shape of the scleral flap in PDS is a rectangle, whereas in PC it is ellipsoid. Suturing a flap to the sclera can create a redistribution of mechanical tensions. The possible effect on astigmatism concerning the shape of the scleral flap was previously discussed in the literature. Tanito et al.^21^ suggested that that the triangular flap may characterize better readaptation than a rectangular one, thus creating little probability for an unsupported corneal edge. The same situation can be addressed to PC, as there are relatively many tight sutures along the whole edge of the flap, which enables good adaptation.

Another aspect is the cauterization of the scleral wound, which may contract the tissue, then cause the reshaping of the sclera. In PDS cauterization is used routinely, whereas in PC it should be avoided when possible. Cauterization closes superficial distal outflow pathways, which are indirectly a target in this surgery^22^.

Well-functioning filtering blebs in PDS rarely cause dysesthesia^23^. When the surgery achieves a stable state, the filtering blebs are relatively flat and usually do not cause vertical steepening of the cornea. In contrary to trabeculectomy, where with the rule astigmatism is often induced^24–26^ followed by against the rule shift.

Induction of postoperative astigmatism may be influenced by various intraocular pressure levels, especially when the hypotony is present, the eye is more vulnerable to shape changes. It plays a vital role in penetrating surgeries, with a higher risk of a long-lasting profound hypotony. In our study, the mean IOPs were lower in PC, which was statistically significant, but we had no cases of long-standing hypotony, which would persist until a 6-month follow-up. A regression analysis was calculated however, it showed no relationship between astigmatism values and IOP levels.

Additionally, we analysed data for separate right and left eyes. However, no differences were found between eyes for single surgery (PC and PDS) in all time points (results are not included in the paper).

During the first postoperative month, 95% of PDS patients received 5-FU subconjunctival injections, ranging from 1 to 10, with the mean number 3.75 injections per person. 5-FU is a well-known antimetabolite to improve success in bleb-dependent procedures. However, it is also known to induce intermittent irregular astigmatism. However, in a longer perspective, after 6 and 24 months, we did not see the refractive difference.

The most important conclusion of the conducted analysis is that both non penetrating surgeries, do not induce significant refractive change. This is true for the within-group analysis as well between the groups.

This study although prospective, randomized has its limitations. The surgery itself combined cataract extraction with glaucoma and cataract surgery may influence the outcomes. At baseline, there is a lenticular component of astigmatism, whereas postoperatively IOL component. This analysis was based on AKR data, which evaluates whole astigmatism, not only corneal changes. On the other hand, it presents a real-life data.

The study is a single-center study and the sample number is limited.
